# Endothelin-2-Mediated Protection of Mutant Photoreceptors in Inherited Photoreceptor Degeneration

**DOI:** 10.1371/journal.pone.0058023

**Published:** 2013-02-28

**Authors:** Alexa N. Bramall, Michael J. Szego, Laura R. Pacione, Inik Chang, Eduardo Diez, Pedro D'Orleans-Juste, Duncan J. Stewart, William W. Hauswirth, Masashi Yanagisawa, Roderick R. McInnes

**Affiliations:** 1 Program in Developmental Biology, The Research Institute, The Hospital for Sick Children, Toronto, Ontario, Canada; 2 Department of Molecular Genetics, University of Toronto, Toronto, Ontario, Canada; 3 Lady Davis Research Institute, Jewish General Hospital, McGill University, Montreal, Quebec, Canada; 4 Department of Molecular Genetics and Howard Hughes Medical Institute, University of Texas Southwestern Medical Center, Dallas, Texas, United States of America; 5 Department of Anatomy and Cell Biology, Faculty of Medicine, Université de Sherbrooke, Sherbrooke, Quebec, Canada; 6 The Regenerative Medicine Program, Ottawa Hospital Research Institute, University of Ottawa, Ottawa, Ontario, Canada; 7 Department of Ophthalmology, College of Medicine, University of Florida, Gainesville, Florida, United States of America; Morehouse School of Medicine, United States

## Abstract

Expression of the *Endothelin-2 (Edn2)* mRNA is greatly increased in the photoreceptors (PRs) of mouse models of inherited PR degeneration (IPD). To examine the role of *Edn2* in mutant PR survival, we generated *Edn2^−/−^* mice carrying homozygous *Pde6b^rd1^* alleles or the *Tg(RHO P347S)* transgene. In the *Edn2^−/−^* background, PR survival increased 110% in *Pde6b^rd1/rd1^* mice at post-natal (PN) day 15, and 60% in *Tg(RHO P347S)* mice at PN40. In contrast, PR survival was not increased in retinal explants of *Pde6b^rd1/rd1^*; *Edn2^−/−^* mice. This finding, together with systemic abnormalities in *Edn2^−/−^* mice, suggested that the increased survival of mutant PRs in the *Edn2^−/−^* background resulted at least partly from the systemic EDN2 loss of function. To examine directly the role of EDN2 in mutant PRs, we used a scAAV5-*Edn2* cDNA vector to restore *Edn2* expression in *Pde6b^rd1/rd1^*; *Edn2^−/−^* PRs and observed an 18% increase in PR survival at PN14. Importantly, PR survival was also increased after injection of scAAV5-*Edn2* into *Pde6b^rd1/rd1^* retinas, by 31% at PN15. Together, these findings suggest that increased *Edn2* expression is protective to mutant PRs. To begin to elucidate *Edn2*-mediated mechanisms that contribute to PR survival, we used microarray analysis and identified a cohort of 20 genes with >4-fold increased expression in *Tg(RHO P347S)* retinas, including *Fgf2*. Notably, increased expression of the FGF2 protein in *Tg(RHO P347S)* PRs was ablated in *Tg(RHO P347S)*; *Edn2^−/−^* retinas. Our findings indicate that the increased expression of PR *Edn2* increases PR survival, and suggest that the *Edn2*-dependent increase in PR expression of FGF2 may contribute to the augmented survival.

## Introduction

IPDs are genetically heterogeneous disorders characterized by the progressive death of mutant PRs. Although more than 160 IPD-associated genes have been identified in humans, with many of these mutations modeled in mouse, relatively few studies have examined the biochemical mechanisms that promote or resist death in the mutant PR [1], [2]. Factors shown to promote the survival of mutant or injured PRs include IL-6 cytokines [3], [4], [5], [6], [7], STAT3 [8], and neurotrophic factors including FGF2 [9], [10], [11], [12], [13]. In contrast, other molecules including GFAP and vimentin [14], [15], complement factor D [16], TNFα [17] and poly-ADP-ribose polymerase-1 [18] have been shown to contribute to the death of mutant or injured PRs.

One gene whose PR expression is strongly induced by PR mutations is *Edn2* [19]. Retinal *Edn2* transcripts are up-regulated in multiple models of photoreceptor degeneration [19], [20], [21], as well as in other retina stresses including, for example, retinal detachment [19], [22] and retinal hypoxia [23] amongst others. These observations suggest that the increased expression of *Edn2* may be a general response to retinal insult.

EDN2 is a vasoactive peptide that binds to two G-protein coupled receptors, EDNRA and EDNRB, with equal affinity [24]. EDNRB is expressed in both Müller glia [19] and horizontal cells [25] in neural retina, whereas EDNRA is present in bipolar dendrites [25]. Both receptors are detected in choroidal and retinal vessels [26].

The biological roles of EDN2 are the least well-characterized of the three endothelin family members [27]. EDN2 has been found to participate in macrophage chemoattraction in breast tumor cell invasion [28], [29], keratinocyte differentiation [30], and oviductal contraction during ovulation [31], [32]. Within the context of PR injury, the effect of the increased expression of *Edn2* on PR survival is unclear. Whereas the intravitreal administration of an EDNRB antagonist increased PR death in one IPD model [5] and reduced the protective effects of Norrin in light damage [33], the subcutaneous administration of a dual EDNRA-EDNRB receptor antagonist after light damage reduced PR layer staining for cleaved caspase-3, a cell death marker [25].

We used genetic analyses to determine whether the increased expression of *Edn2* in mutant PRs is a pathogenic or a pro-survival response, in two well-defined mouse models of IPDs, mice carrying a mutant human rhodopsin transgene (*Tg(RHO P347S)*) [34] associated with human PR degeneration, and *Pde6b^rd1/rd1^* mice, with a mutation in the β subunit of the PR-specific phosphodiesterase-6 (PDE-6) gene [35]. We over-expressed *Edn2* in mutant PRs and found that it plays a protective role in one model of IPD. We also provide evidence consistent with this protection being at least partly mediated by FGF2, a known neuroprotective factor [9].

## Results

### Increased expression of the *Edn2* mRNA and peptide in mutant PRs

**Figure 1:**
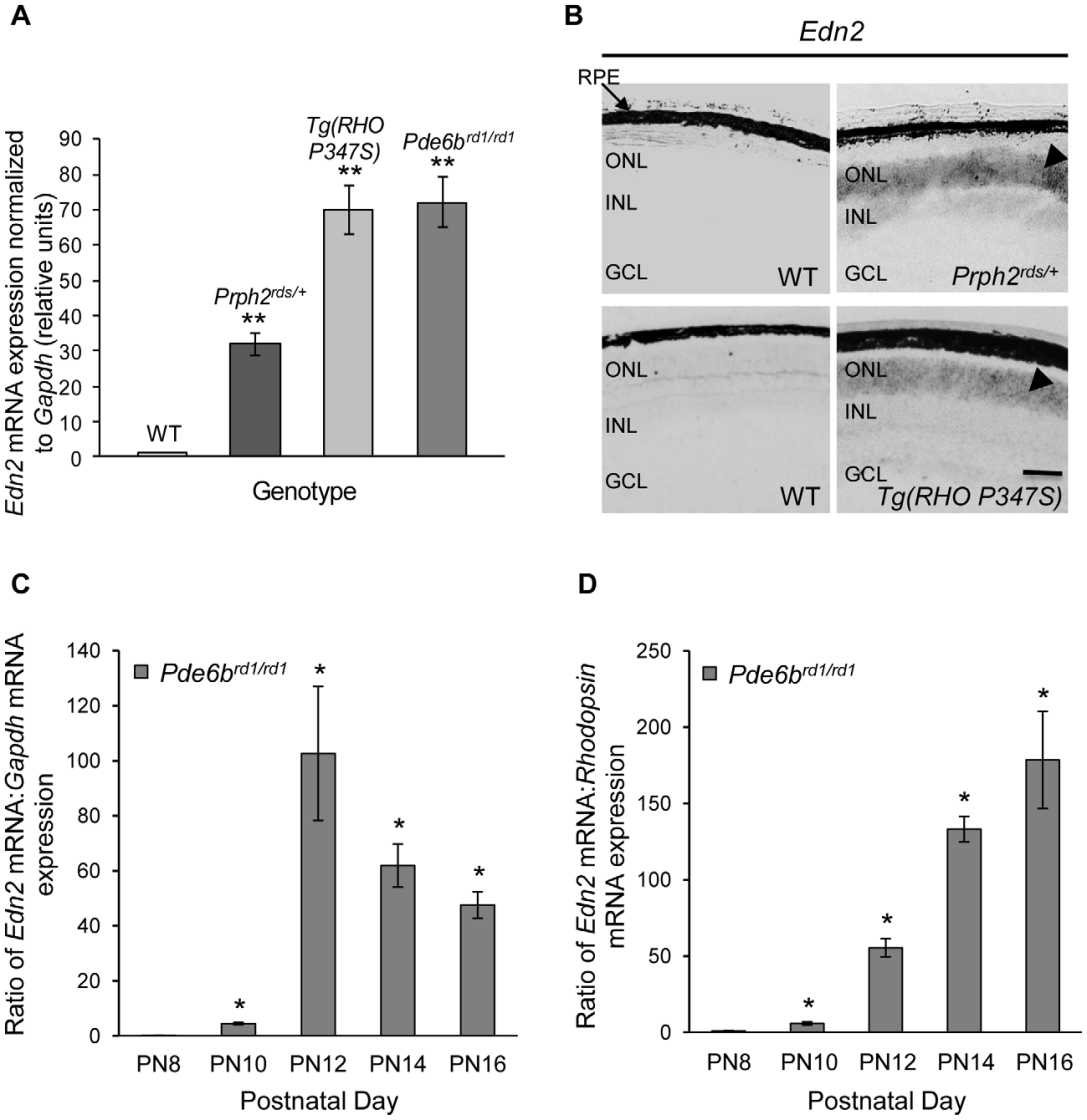
Retinal *Edn2* mRNA is increased in several models of IPD. (A) qRT-PCR assays of the *Edn2* mRNA. Edn2 was increased 32-fold, 70-fold, and 72-fold in the *Prph2^rds/+^* (7 weeks), *Tg(RHO P347S)* (3 weeks) and *Pde6b^rd1/rd1^* (PN12) models of IPD, respectively (^**^n = 3; Student's t-test p<0.005). At the three time points chosen, >40% of the PR population remained. *Edn2* mRNA expression was assigned a value of 1 in WT retinas, to calculate its relative expression in the IPD models. qRT-PCR values were normalized to the mRNA expression of *Gapdh*. (B) *In situ* hybridization of *Edn2* mRNA in *Prph2^rds/+^* and *Tg(RHO P347S)* retinas. *Edn2* transcripts were undetectable in WT retina, but were detected exclusively in the ONL of the mutant PRs (arrowheads) in *Prph2^rds/+^* and *Tg(RHO P347S)* retinas. (C,D) Temporal expression of *Edn2* transcripts during PR degeneration in *Pde6b^rd1/rd1^* retinas, relative to WT *Edn2* mRNA expression at PN12 and normalized to *Gapdh* mRNA (C), or rhodopsin mRNA (D). *Edn2* mRNA was not significantly increased until PN10 (^*^n = 3; p<0.05 vs. WT). RPE, retinal pigment epithelium; ONL, outer nuclear layer; INL, inner nuclear layer; GCL, ganglion cell layer. Error bars indicate SEM; scale bar = 10 µm.

To confirm that *Edn2* transcripts are up-regulated in the *Prph2^rds/+^*, *Tg(RHO P347S)*, and *Pde6b^rd1/rd1^* mouse models of IPD we studied, we measured retinal *Edn2* mRNA abundance. As reported by Rattner and Nathans in other models [19], we found the *Edn2* mRNA to be greatly increased vs. wild-type (WT) controls in *Prph2^rds/+^* (32-fold), *Tg(RHO P347S)* (70-fold), and *Pde6b^rd1/rd1^* (72-fold) retinas, using qRT-PCR (for each model n = 3; p<0.005) (Fig. 1A).

We also confirmed, by *in situ* hybridization using *Prph2^rds/+^* and *Tg(RHO P347S)* mice, the observation of Rattner and Nathans [19] that the increase in the *Edn2* mRNA is PR-specific (Fig. 1B). To determine if *Edn2* mRNA up-regulation is a leading or lagging indicator of PR death, we examined the temporal up-regulation of *Edn2* mRNA in *Pde6b^rd1/rd1^* retinas by qRT-PCR (Fig. 1C,D). The abundance of the *Edn2* transcript in *Pde6b^rd1/rd1^* retinas at PN8, measured as the ratio of *Edn2* mRNA:Gapdh mRNA expression, was comparable to the expression of *Edn2* transcripts in WT retinas at all time points examined (data not shown). By PN10, however, a statistically significant increase in *Edn2* transcripts was detected in *Pde6b^rd1/rd1^* retinas (Fig. 1C,D)(^*^n = 3, p<0.05 vs. WT retinas; at this age, pycnotic nuclei are present in this mutant [36]). The decrease in *Edn2* mRNA expression with time (Fig. 1C) is likely to reflect the decreasing number of PRs in the *Pde6b^rd1/rd1^* retina, since the mRNA expression of *Edn2* relative to rhodopsin increases with time (Fig. 1D), indicating that the expression of the *Edn2* mRNA per PR increased as the number of PRs progressively decreased. *Edn2* transcripts remained substantially up-regulated as late as PN18 (data not shown).

To determine whether the increase in *Edn2* transcripts in mutant retinas is accompanied by increased levels of EDN2 peptide, we quantified EDN2 in *Prph2^rds/+^* retinas by radioimmunoassay after separating the EDN2 peptide from EDN1 and EDN3 by HPLC. The abundance of EDN2 was below the detection limit in WT retinas (<0.05 fmol/retina; n = 10 retinas), making it impossible to determine the maximum fold increase in EDN2 mutant retinas. However, 0.15 fmol/retina (n = 10 retinas) of EDN2 was detected in *Prph2^rds/+^* retinas, indicating that the EDN2 peptide is increased by a minimum of three-fold in *Prph2^rds/+^* retinas.

### Retinal expression of components of endothelin signaling biology

To evaluate whether the large increase in *Edn2* expression in mutant PRs was accompanied by increases in other components of endothelin biology, we first quantified the expression of the mRNAs for *Edn1*, *Edn3, EdnrA*, and *EdnrB* in WT and mutant retinas (Fig. S1). Overall, there were no significant differences at PN21 between *Edn2^+/+^*, *Edn2^−/−^*, *Tg(RHO P347S);Edn2^+/+^* and *Tg(RHO P347S);Edn2^−/−^* retinas in the expression of these genes (all n = 3, p>0.05) (Fig. S1), with one exception: the mRNA of *EdnrA* in *Tg(RHO P347S);Edn2^−/−^* was modestly increased, by 2.3-fold, compared to *Edn2^+/+^* retinas (n = 3, p<0.05) (Fig. S1).

**Figure 4:**
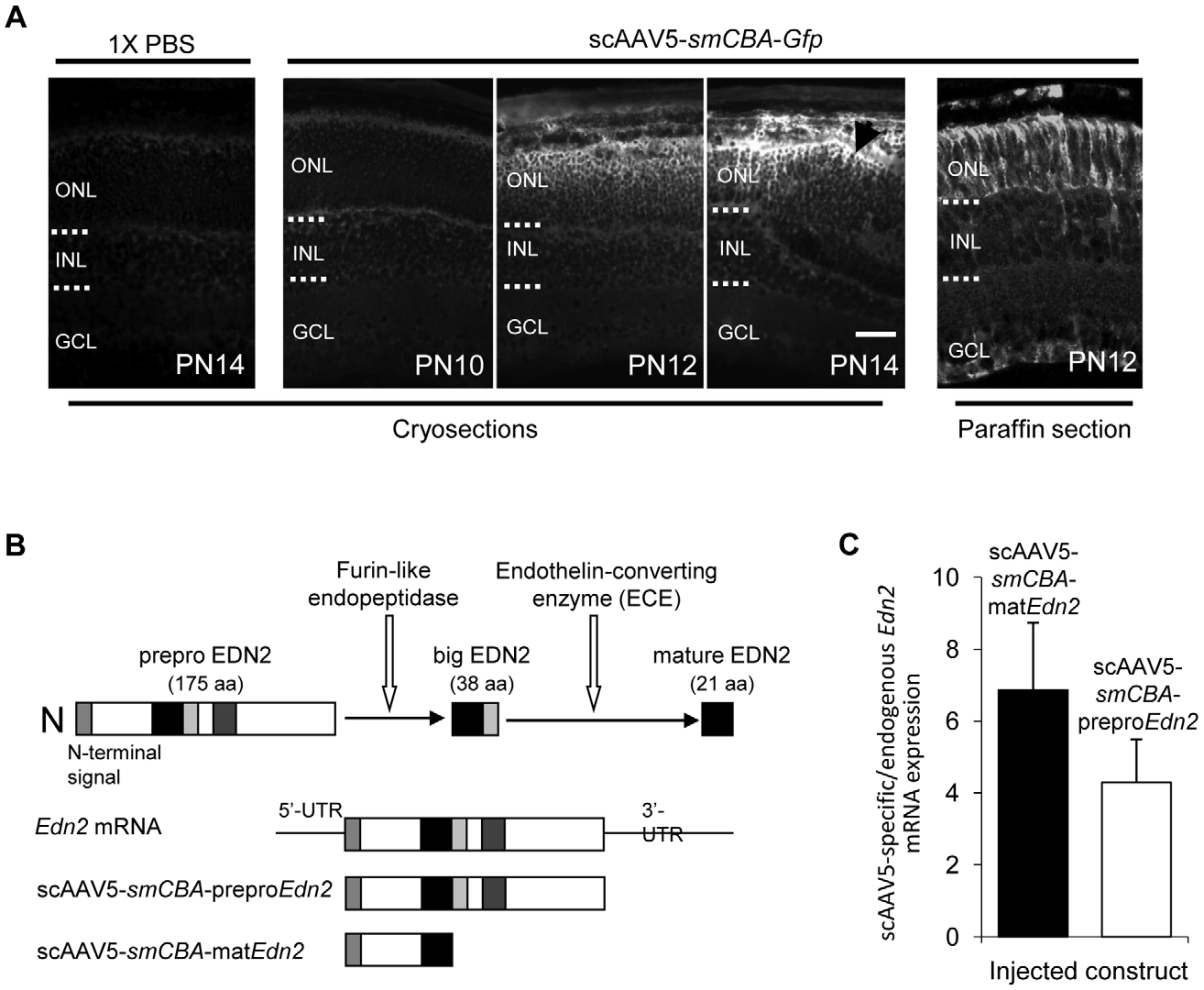
Expression of GFP and *Edn2* from subretinally injected scAAV5. (A) The temporal and spatial expression of GFP in WT retinas injected subretinally with 1X PBS or scAAV5-smCBA-*Gfp* at PN8 and evaluated at PN10, PN12, and PN14 by GFP immunofluorescence (A, Panels 1–4). Significant GFP staining was not observed until PN12, with stronger staining at PN14, especially in the vicinity of the subretinal injection site (arrow). The spatial expression of GFP in WT retinas injected with scAAV5-smCBA-*Gfp* was also evaluated in paraffin sections at PN12 (A, Panel 5). GFP expression was observed predominantly in the ONL and RPE; sporadic expression of GFP in Müller cells was also observed in paraffin sections. (Bar = 25 µm.) (B) Schematic of the EDN2 cleavage events required to produce the mature EDN2 peptide. EDN2 is first produced as prepro EDN2 (175 aa) which is rapidly processed by furin-like endopeptidases to yield big EDN2 (38 aa). Big EDN2 must then be cleaved by an endothelin-specific converting enzyme (ECE) to produce the 21 a.a. mature EDN2 peptide that can bind to endothelin receptors (figure adapted from [68]). The regions of the *Edn2* mRNA corresponding to the cDNAs cloned into the scAAV5-*smCBA*-prepro*Edn2* and scAAV5-*smCBA*-mat*Edn2* vectors are shown. (C) Expression of scAAV5-derived *Edn2* mRNA in *Pde6b^rd1/rd1^* retinas at PN12 after injection of the scAAV5-*smCBA*-prepro*Edn2* and scAAV5-*smCBA*-mat*Edn2* constructs at PN8. scAAV5-derived *Edn2* mRNA expression values are shown relative to the levels of endogenous *Edn2* mRNA (from the same retina) and all values were normalized to *Gapdh*. scAAV5-prepro*Edn2* transcripts were increased between 1.7 and 7.2-fold (n = 4; average 4.3-fold) over endogenous *Edn2* mRNA, while scAAV5-mat*Edn2* transcripts increased between 2.5 to 11.3-fold over the endogenous *Edn2* mRNA (n = 4; average 6.9-fold). ONL, outer nuclear layer; INL, inner nuclear layer; GCL, ganglion cell layer. Error bars indicate SEM.

EDN2 is processed from a prepro peptide that undergoes sequential cleavage events to generate mature EDN2 (mat EDN2) (Fig. 4B). To determine where endothelin-converting enzyme 1 (ECE-1), an enzyme implicated in the processing of big EDN2 to mature EDN2 [37], is expressed in retina, we used immunofluorescence staining. We found that ECE-1 was expressed in Müller cell bodies and radial fibres (Fig. S2A), as shown by comparison with the Müller cell-specific marker glutamine synthetase [38].

To determine whether the expression pattern of the EDNRA and EDNRB receptors was altered in *Pde6b^rd1/rd1^* retinas, in the presence or absence of *Edn2* expression, we used immunofluorescence staining (Fig. S2B). In mice of all four genotypes, EDNRA expression was low, with only scattered staining in the choroid, OPL, INL and GCL, which may represent retinal microglia (42) that migrate to the mutant PRs in the ONL [39]. There was no significant difference in EDNRA staining in *Pde6b^rd1/rd1^*; *Edn2^+/+^* vs. *Pde6b^rd1/rd1^*; *Edn2^−/−^* retinas (Fig. S2B).

EDNRB expression in WT retina was seen predominantly in the outer plexiform layer (OPL) as well as in cells, possibly astrocytes, in the GCL (Fig. S2B). In the *Pde6b^rd1/rd1^* retina, EDNRB expression in the *Pde6b^rd1/rd1^* retina was increased throughout Müller cell radial fibres, with stronger staining in the inner limiting membrane (ILM), which contains the end feet of Müller cells. In contrast to the OLM staining observed in light-damaged retinas [19], no outer limiting membrane (OLM) staining of ENDRB was detected in the *Pde6b^rd1/rd1^* retina. EDNRB expression remained increased in the absence of EDN2 in the *Pde6b^rd1/rd1^*; *Edn2^−/−^* retina (Fig. S2B), indicating that the up-regulation of EDNRB expression in the mutant retina is not EDN2-dependent.

A simple model consistent with the expression pattern of the endothelin receptors and converting enzyme in the PR mutant retina is therefore that big EDN2 produced in mutant PRs is released into the PR extracellular space, and converted to mat EDN2 by the extracellular moiety of ECE-1 [40] located on Müller cell radial fibres that surround the PRs. The mat EDN2 could then bind to EDNRB, also located on Müller cell radial fibres.

### EDN2 loss of function increases PR survival *in vivo*, but not *ex vivo*

**Figure 2:**
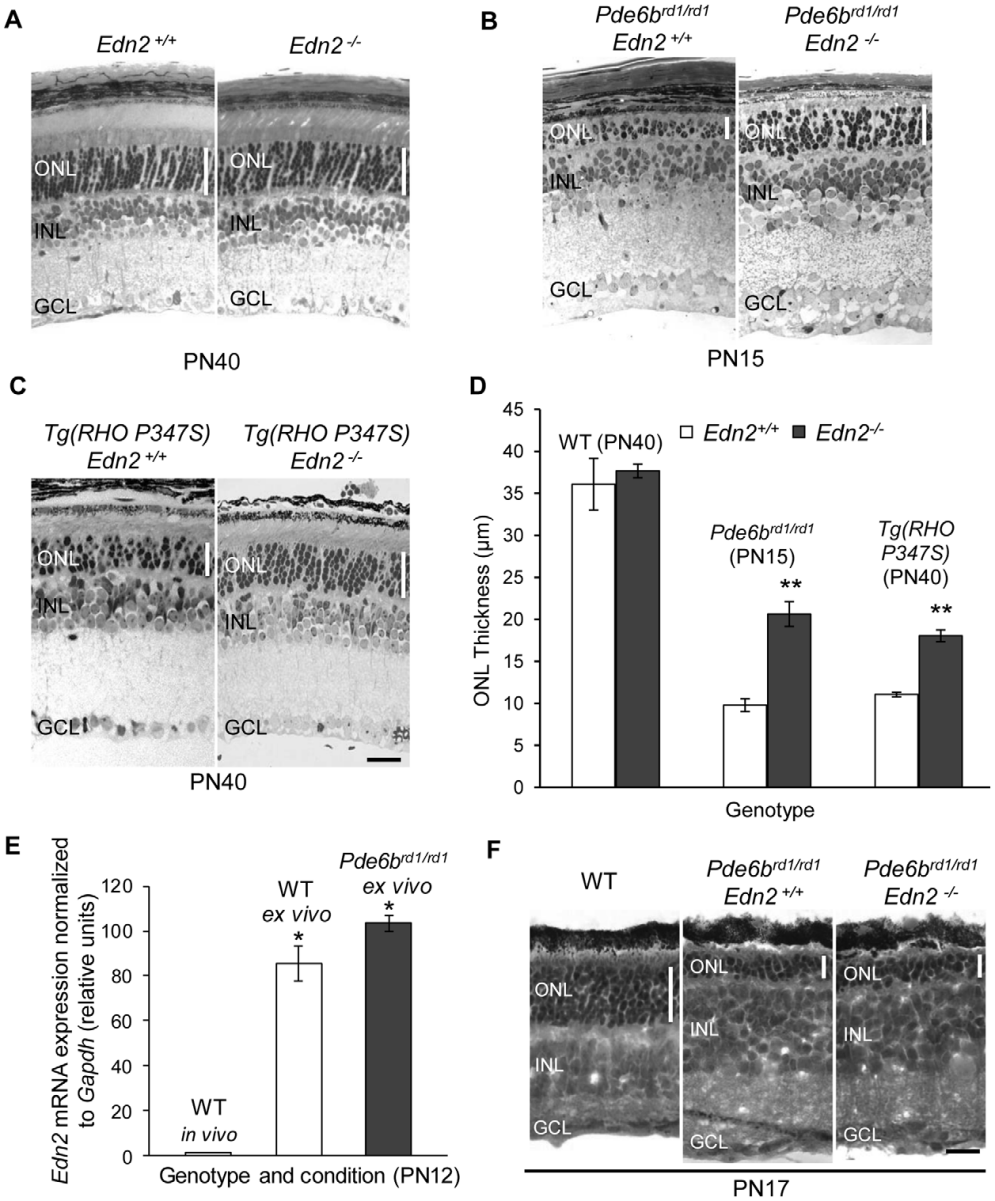
The effect of EDN2 loss on mutant PR survival *in vivo* and in retinal explants. (A) At PN40, the histology and the thickness of the ONL (n = 5;p>0.05) was normal in toluidine-blue stained *Edn2^+/+^* and *Edn2^−/−^* retinas. (B,C) The loss of EDN2 in *Tg(RHO P347S)* retinas resulted in a mean 63% increase in ONL thickness at PN40 (n = 6; p<0.005) (C) and a mean 110% increase in ONL thickness in *Pde6b^rd1/rd1^* retinas at PN15 (n = 6; p<0.005) (B). (D) ONL thickness in WT, *Pde6b^rd1/rd1^* and *Tg(RHO P347S)* retinas in mice expressing or lacking EDN2 (^**^p<0.005). (E) qRT−PCR assays of the *Edn2* mRNA, normalized to *Gapdh* mRNA, in *in vivo* WT, WT explants and *Pde6b^rd1/rd1^* explants (n = 3;^*^p<0.05). Values were compared to the mean *Edn2* mRNA levels in WT *in vivo* samples (arbitrarily given a value of 1). *Edn2* transcripts were significantly increased in WT as well as *Pde6b^rd1/rd1^* explants at PN12 following retinal dissection at PN7, likely as a result of dissection-induced mechanical stress. (F) WT retinal explants cultured *ex vivo* from PN7-PN17 had an average of 7–8 rows of PR nuclei at PN17 (n = 10 retinas, one representative shown). Owing to artifacts in frozen sections, the number of nuclei, instead of ONL thickness, was assessed in retinal explants. The absence of EDN2 in *Pde6b^rd1/rd1^*; *Edn2^−/−^* retinal explants did not increase PR survival. Both *Pde6b^rd1/rd1^* and *Pde6b^rd1/rd1^*; *Edn2^−/−^* explants cultured from PN7-PN17 had an average of 3 rows of PR nuclei at PN17 (n = 4;p>0.05, one representative shown) (H&E staining). ONL, outer nuclear layer; INL, inner nuclear layer; GCL, ganglion cell layer. (Black bar = 25 µm in A–C, and F). Error bars indicate SEM.

To determine whether EDN2 promotes, resists, or has no influence on PR survival in IPDs, we used *Edn2^−/−^* mice to generate *Tg(RHO P347S)*; *Edn2^−/−^* and *Pde6b^rd1/rd1^*; *Edn2^−/−^* animals. We first determined that EDN2 is not required for normal retinal or PR formation or survival by examining the morphology of adult *Edn2^−/−^* retinas. No significant difference in gross morphology, or in ONL thickness (n = 5; p>0.05), was observed between WT and *Edn2^−/−^* retinas at PN40 (Fig. 2A). *Edn2^−/−^* mice were born at normal Mendelian ratios but exhibited runting by PN7 and a survival rate of only 25% at PN20 (unpublished observations). *Edn2^+/−^* mice displayed no overt phenotype. To allow observation of the effects of EDN2 loss in the slower degenerating *Tg(RHO P347S)* retinas, we were able to extend the lifespan of *Edn2^−/−^* mice to a maximum of PN50 by using a liquid diet and daily subcutaneous injections of normal saline to maintain fluid and electrolyte balance.

Remarkably, the loss of EDN2 function led to dramatically increased PR survival in both *Pde6b^rd1/rd1^* and *Tg(RHO P347S)* mice. At PN15, the thickness of the ONL increased by 110% in *Pde6b^rd1/rd1^*; *Edn2^−/−^* vs. *Pde6b^rd1/rd1^*; *Edn2^+/+^* retinas at PN15 (n = 6; p<0.05) (Fig. 2B), and by 63% in *Tg(RHO P347S)*; *Edn2^−/−^* vs. *Tg(RHO P347S); Edn2^+/+^* retinas at PN40 (n = 6; p<0.05) (Fig. 2C). The increased PR survival was not due to a compensatory change in the expression of *Edn1* and *Edn3* mRNAs due to the absence of EDN2 in *Edn2^−/−^* retinas, because the abundance of these two mRNAs in *Edn2^−/−^* retinas was not significantly different from WT (Fig. S1). In summary, these findings indicate that the systemic loss of EDN2 in mouse models of IPD strongly protects PRs from death (Fig. 2D).

To separate the effects of increased retinal *Edn2* in *Pde6b^rd1/rd1^* retinas from potentially confounding systemic *Edn2^−/−^* phenotypes, such as hypoxia (see below), we examined the impact of EDN2 loss on PR death in an *ex vivo* retinal explant system. *Pde6b^rd1/rd1^* retinas were used for the retinal explant assays because they recapitulate, in the explant, the PR death seen in the *Pde6b^rd1/rd1^* mouse (40), manifesting substantial PR death well before the explant is no longer viable (at four weeks in culture). Retinas were removed from mice sacrificed at PN7, well before the onset of PR degeneration [36] and, owing to a slightly slower rate of degeneration *ex vivo*, examined at PN17. qRT-PCR measurements of *Edn2* transcripts in retinal explants showed that *Edn2* mRNA is induced both in WT and *Pde6b^rd1/rd1^* retinal explants at PN12 (Fig. 2E). The induction of *Edn2* mRNA in WT explants was anticipated, since *Edn2* transcripts are induced as a result of retinal detachment [19], and retinal dissections are associated with substantial mechanical stress. WT retinas cultured from PN7 until PN17 had 7–8 rows of PRs at PN17 (n = 6), as expected [41] (Fig. 2F). In contrast to the increased survival of mutant PRs observed in *Pde6b^rd1/rd1^*; *Edn2^−/−^* and *Tg(RHO P347S)*; *Edn2^−/−^* retinas *in vivo*, the absence of EDN2 had no effect on PR survival in *Pde6b^rd1/rd1^*; *Edn2^−/−^* retinal explants: both *Pde6b^rd1/rd1^* and *Pde6b^rd1/rd1^*; *Edn2^−/−^* explants had 4–5 rows of PRs at PN16 (data not shown) and 2–3 rows of PRs at PN17 (Fig. 2F) (n = 6; p>0.05). Consequently, the fact that the rate of PR death in *Pde6b^rd1/rd1^*; *Edn2^−/−^* retinas is slowed *in vivo* but not in retinal explants suggests the possibility that the increased PR survival seen *in vivo* may be partly or entirely due to extra-ocular effects of the loss of EDN2 function.

### Hypoxia as one mechanism for PR protection in *Pde6b^rd1/rd1^*; *Edn2^−/−^* and *Tg(RHO P347S)*; *Edn2^−/−^* mice

**Figure 3:**
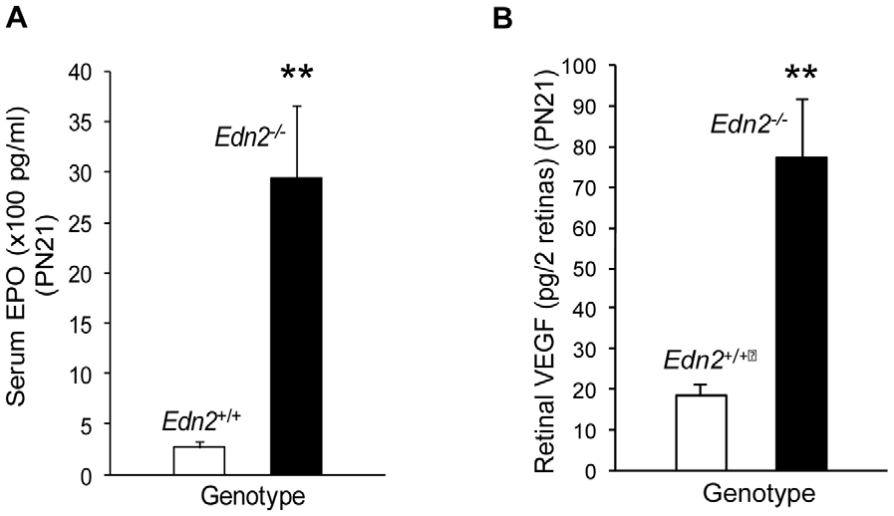
Systemic erythropoietin (EPO) and retinal vascular endothelial growth factor (VEGF) is increased in *Edn2^−/−^* mice. At PN21, serum EPO was increased 11-fold in Edn2^−/−^ vs. Edn2^+/+^ mice (n = 7; p<0.005) (A) and retinal VEGF was increased 4-fold (n = 4; p<0.005) (B). EPO and VEGF were both measured using ELISA assays. Error bars indicate SEM.

To identify possible systemically-mediated causes of the increased *in vivo* survival of PRs in *Tg(RHO P347S)*; *Edn2^−/−^* and *Pde6b^rd1/rd1^*; *Edn2^−/−^* retinas, we examined *Edn2^−/−^* mice for gross morphological abnormalities. Among other phenotypes, we found that *Edn2^−/−^* mice have defective lungs leading to systemic hypoxia (unpublished data). The hypoxia was associated with an 11-fold increase in erythropoietin (EPO) levels at PN21 (n = 7; p<0.05)(Fig. 3A). Moreover, vascular endothelial growth factor (VEGF) levels in *Edn2^−/−^* retinas were 4.2-fold elevated (n = 4; p<0.05), an increase consistent with the presence of retinal hypoxia (Fig. 3B). The presence of systemic and probable retinal hypoxia in *Edn2^−/−^* mice suggests that hypoxia may be at least partially responsible for the increased *in vivo* PR survival in *Pde6b^rd1/rd1^*; *Edn2^−/−^* and *Tg(RHO P347S)*; *Edn2^−/−^* retinas, since we have previously demonstrated that hypoxia increases PR survival in *Pde6b^rd1/rd1^* retinal explants [42].

### Over-expression of *Edn2* in PRs increases their survival in *Pde6b^rd1/rd1^* mice

To examine directly the role of increased *Edn2* expression in mutant PRs, we introduced an *Edn2* cDNA into *Pde6b^rd1/rd1^* and *Pde6b^rd1/rd1^*; *Edn2^−/−^* retinas using the scAAV5-*smCBA* vector [43]. Due to the low survival rate of *Edn2^−/−^* mice, we performed the scAAV5 experiments in *Pde6b^rd1/rd1^* mice. We initially confirmed the PR expression of this vector by assessing the spatial and temporal expression of GFP from a scAAV5-*smCBA-Gfp* (AAV-*Gfp*) vector in frozen sections (Fig. 4A, panels 1–4). Using paraffin sections, GFP staining was observed in PR nuclei, as reported by others [43] (Fig. 4A, panel 5). AAV-*Gfp* was injected subretinally on PN8 and GFP expression examined on PN10, PN12 and PN14 (Fig 4A). PR expression of GFP was evident throughout the ONL and retinal pigment epithelium (RPE), with the highest expression adjacent to the injection site (Fig 4A, arrow) and in half of the retina extending from the optic disc to periphery (data not shown). Minimal GFP staining was seen two days post-injection, with significantly stronger expression at PN12 and PN14 (Fig. 4A), an increase comparable to the observed temporal increase in the endogenous *Edn2* transcript in *Pde6b^rd1/rd1^* retinas (Fig. 1C,D). In subsequent studies, we therefore introduced the AAV vectors at PN8, to assure strong expression by PN12 and later.

To establish that robust retinal expression of a cDNA encoding mature EDN2 could be obtained from a scAAV5-*smCBA*-mat*Edn2* vector (AAV-mat*Edn2*), we injected it into *Pde6b^rd1/rd1^* retinas at PN8. At PN12, expression of the mature *Edn2* mRNA was 2.5- to 11.3-fold greater (mean 6.9-fold; n = 4) than the level of the endogenous *Edn2* mRNA in *Pde6b^rd1/rd1^* retinas (Fig. 4C, left bar). Similarly, we quantified the expression of a prepro *Edn2* cDNA from an scAAV5-*smCBA*-prepro*Edn2* (AAV-prepro*Edn2*) vector. Any difference between outcomes in PR survival using the mat*Edn2* vs. the prepro*Edn2* vectors could suggest that the processing of prepro EDN2 to mat EDN2 is rate-limiting. We found that expression of the prepro *Edn2* mRNA was 1.7- to 7.2-fold (average 4.3-fold; n = 4) greater than the level of the endogenous *Edn2* mRNA in *Pde6b^rd1/rd1^* retinas (Fig. 4C, right bar). In summary, in *Pde6b^rd1/rd1^* retinas both the AAV-mat*Edn2* and the AAV-prepro*Edn2* vectors expressed their respective mRNAs at levels on average 4.3- to 6.9-fold above the expression level of the native *Edn2* mRNA.

**Figure 5:**
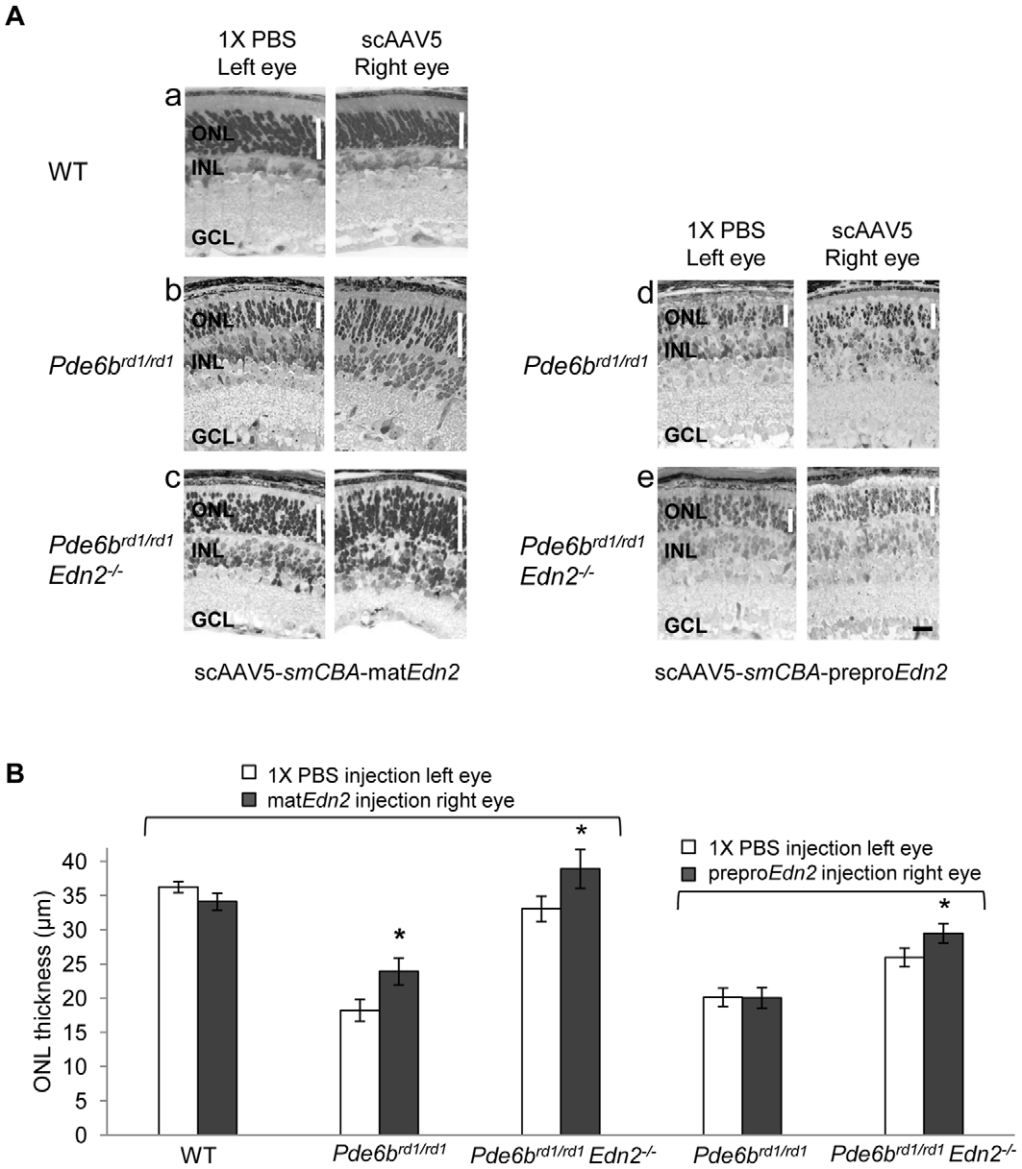
scAAV5-mediated transfer of the mature *Edn2* cDNA at PN8 increases mutant PR survival. (A) Micrographs showing ONL thickness in individual retinas at PN14–15. (a) Injection of the scAAV5-*smCBA*-mat*Edn2* vector into WT retinas at PN8 did not alter retinal morphology at PN15. (b) Injection of the scAAV5-*smCBA*-mat*Edn2* vector at PN8 increased PR ONL thickness of *Pde6b^rd1/rd1^* retinas by 31% (n = 9; ^*^p<0.05) at PN15, and (c) of *Pde6b^rd1/rd1^*; *Edn2^−/−^* retinas at PN14 by 18% (n = 5; ^*^p<0.05). (d) In contrast, injection of the scAAV5-*smCBA*-prepro*Edn2* vector at PN8 had no effect on PR ONL thickness of *Pde6b^rd1/rd1^* retinas (n = 6;p>0.05) at PN15, although (e) this vector improved PR survival in *Pde6b^rd1/rd1^*; *Edn2^−/−^* retinas by 14% (n = 6; ^*^p<0.05). (B) A bar graph summarizing the effects of AAV vectors expressing *Edn2* cDNAs, injected at PN8, on mutant PR survival in *Pde6b^rd1/rd1^* and *Pde6b^rd1/rd1^*; *Edn2^−/−^* retinas at PN14–15. ONL, outer nuclear layer; INL, inner nuclear layer; GCL, ganglion cell layer. (Black bar = 25 µm). Error bars indicate SEM.

To examine the role of the increased PR expression of *Edn2* on PR survival in IPD, we augmented the endogenous increase in *Edn2* expression in PRs in *Pde6b^rd1/rd1^* mice using AAV vectors (Fig. 5). We used 1X PBS injection as the control injection in the contralateral eye, because when we compared ONL thickness in 1X PBS injected retinas to AAV-*Gfp* injected *Pde6b^rd1/rd1^* retinas, we observed no difference. The introduction of AAV-mat*Edn2* at PN8 in WT retinas did not alter PR morphology or survival at PN15 (n = 6; p>0.05) (Fig. 5A(a)). Remarkably, the injection of AAV-mat*Edn2* increased retinal ONL thickness in *Pde6b^rd1/rd1^* retinas at PN15 by 31% (n = 9; p<0.05) compared to the injection of 1X PBS in the contralateral eye (Fig. 5A(b)). In contrast, the AAV-prepro*Edn2* vector failed to improve PR survival in *Pde6b^rd1/rd1^* retinas (n = 6; p>0.05) (Fig. 5A(d)), Fig. 5B), suggesting that ECE enzyme sites may be saturated in this context, in which the endogenous increase in *Edn2* expression is combined with the production of prepro *Edn2* from the AAV vector. We also restored the PR expression of *Edn2* in *Pde6b^rd1/rd1^*; *Edn2^−/−^* mice using the AAV vectors. In this instance, the injection of either the mat*Edn2* or prepro*Edn2* vectors increased ONL thickness at PN14 and PN15, by 18% (n = 5; p<0.05) (Fig. 5A(c)) and 14% (n = 6; p<0.05) (Fig. 5A(e)), respectively. The effects of the AAV vectors on PR survival are summarized in Fig. 5B. Overall, these findings provide evidence that the increase in *Edn2* expression is a protective response that increases the survival of mutant PRs, at least in *Pde6b^rd1/rd1^* retinas.

### Evidence that EDN2-mediated up-regulation of FGF2 expression is a survival response

**Figure 6:**
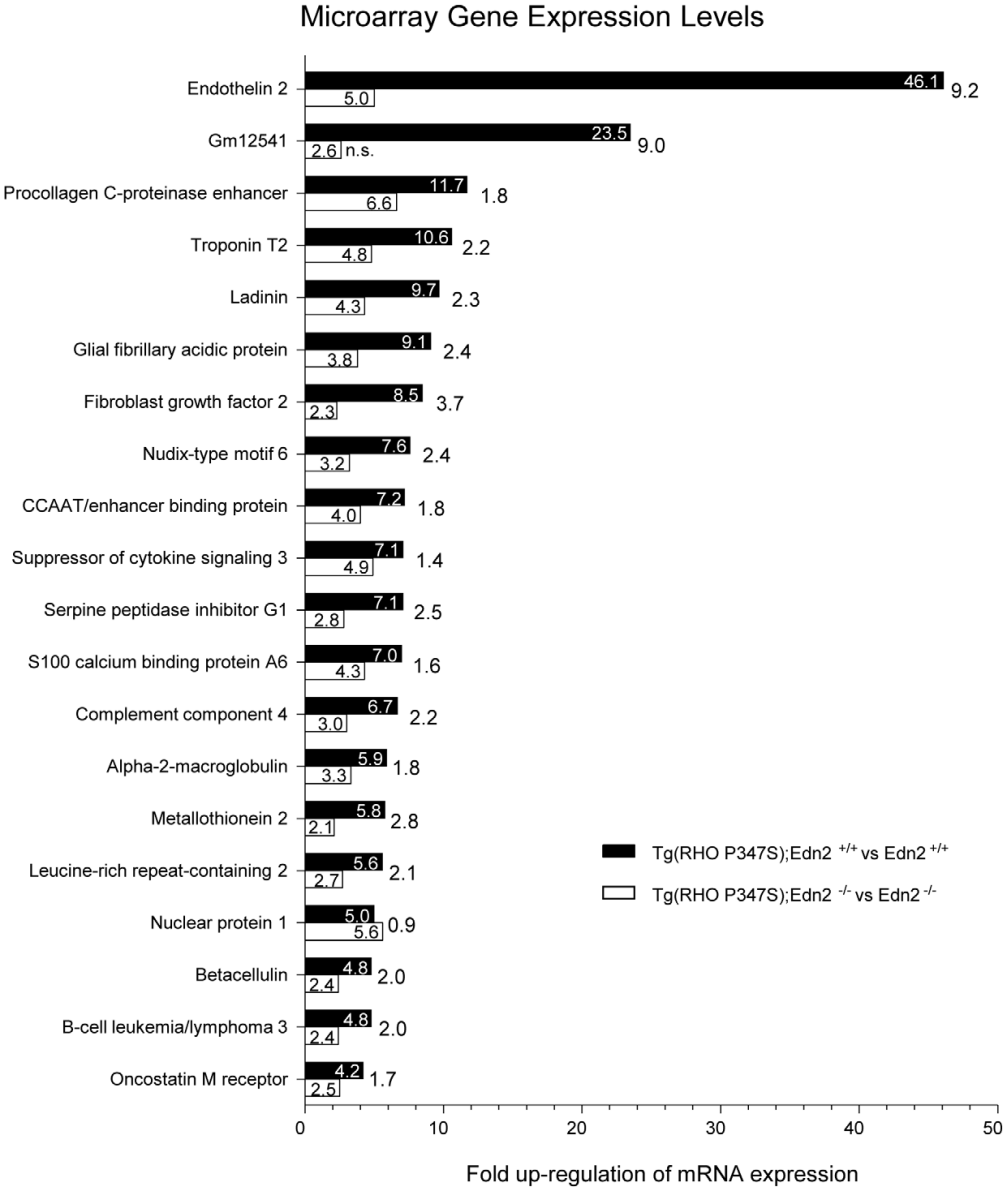
Identification of possible EDN2 regulated genes using microarrays. Gene expression was examined using the Mouse 430_2.0 Affymetrix array (4 arrays/genotype) and the 20 most differentially expressed mRNAs in *Tg(RHO P347S); Edn2^+/+^* vs. *Edn2^+/+^* retinas, and in *Tg(RHO P347S)*; *Edn2^−/−^* vs. *Edn2^−/−^* retinas, at PN21 are shown. All differentially expressed genes in *Tg(RHO P347S)*; *Edn2^+/+^* retinas showed a significant reduction in expression in *Tg(RHO P347S)*; *Edn2^−/−^* retinas, with the exception of *Nuclear protein 1*. The fold reduction is shown to the right of each pair of bars. The detection of *Edn2* transcripts in *Tg(RHO P347S)*; *Edn2^−/−^* retinas is due to the expression of partial *Edn2* mRNAs from the *Edn2^−/−^* locus, in which part of exon 1 and all of exon 2 were replaced with the *Neo* cassette. n.s. =  no statistically significant increase in expression.

To identify retinal genes whose expression is directly or indirectly regulated by EDN2 in the presence of the *Tg(RHO P347S)* mutant allele, we identified mRNAs that were differentially expressed in *Tg(RHO P347S); Edn2^+/+^ vs*. *Edn2^+/+^* retinas, to mRNAs that were differentially expressed between *Tg(RHO P347S)*; *Edn2^−/−^ vs*. *Edn2^−/−^* retinas (Fig. 6). This comparison was used, rather than contrasting the mRNAs of *Tg(RHO P347S); Edn2^+/+^* vs. *Tg(RHO P347S)*; *Edn2^−/−^* retinas, because this latter comparison would fail to distinguish between mRNA changes due the absence of EDN2 alone and indirect changes due to the absence of EDN2 in the presence of the *Tg(RHO P347S)* mutant allele.

The mRNA populations were examined using Mouse 430_2.0 Affymetrix arrays (two retinas from one mouse/array, four arrays/genotype). Retinal mRNA was collected at PN21, three days after the onset (at PN18) of *Edn2* mRNA expression in *Tg(RHO P347S)* retinas (data not shown). Transcripts with a >2.0-fold difference in expression in these two comparisons (p<0.05 using a two class unpaired *t* test) are shown in Figure 6.

The 20 genes whose expression was most induced (>4-fold; p<0.05) in *Tg(RHO P347S); Edn2^+/+^* vs. *Edn2^+/+^* retinas were also induced in *Tg(RHO P347S)*; *Edn2^−/−^* vs. *Edn2^−/−^* retinas (Fig. 6). Notably, the expression of all 20 of these *Tg(RHO P347S)*-induced genes was down-regulated in *Tg(RHO P347S)*; *Edn2^−/−^* retinas (Fig. 6). This finding suggests that EDN2 may have broad effects on the transcription of a cohort of retinal genes (including the 20 genes shown in Fig. 6), a cohort that respond to the presence of a PR mutation, in this instance the *Tg(RHO P347S)* transgene.

**Figure 7:**
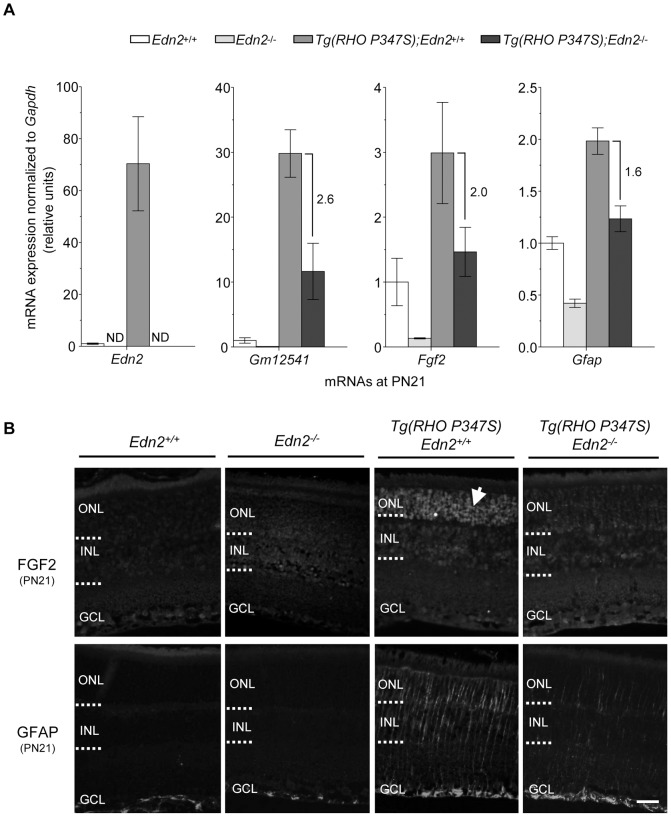
FGF2 expression in *Tg(RHO P347S)* PRs returns to WT levels in the absence of EDN2. (A) qRT-PCR quantification of *Edn2*, *Gm12541*, *Fgf2* and *Gfap* expression in *Tg(RHO P347S)* retinas in the presence or absence of EDN2 function. Bar graphs show the expression of mRNAs in retinas of the indicated genotypes relative to the expression levels seen in *Edn2^+/+^* (WT) retinas. Fold down-regulation in *Tg(RHO P347S)*; *Edn2^−/−^* retinas vs. *Tg(RHO P347S); Edn2^+/+^* retinas is shown to the right of the vertical bars (n = 3;p<0.05 for all mRNAs). All qRT-PCR values were normalized to *Gapdh* mRNA. ND (not detected). (B) Immunostaining for FGF2 showed low levels of expression in all three retinal nuclear layers in *Edn2^+/+^* and *Edn2^−/−^* retinas, but FGF2 expression increased significantly in the PRs of *Tg(RHO P347S); Edn2^+/+^* retinas at PN21 (third top panel). In contrast, FGF2 staining was similar to WT retinas in *Tg(RHO P347S)*; *Edn2^−/−^* retinas and, most notably, from PRs (fourth upper panel). GFAP expression in Müller cells was increased in *Tg(RHO P347S); Edn2^+/+^* retinas (third bottom panel), but reduced in *Tg(RHO P347S)*; *Edn2^−/−^* retinas (fourth bottom panel). GFAP expression in *Tg(RHO P347S)*; *Edn2^−/−^* retinas was higher than in *Edn2^+/+^* retinas. ONL, outer nuclear layer; INL, inner nuclear layer; GCL, ganglion cell layer. (Bar = 25 µm). Error bars indicate SEM.

Of the 20 genes whose expression is most reduced by the loss of EDN2, only *Fgf2* [9], [44], [45] has been shown to increase PR survival in IPDs, and only the loss of *Gfap* has been demonstrated to attenuate PR death due to retinal injury [14]. To investigate the role of *Fgf2* and *Gfap* in the response of the *Tg(RHO P347S)* retina to the loss of EDN2 function, we first used qRT-PCR to confirm and to quantify the decrease in the expression of *Fgf2* and *Gfap* in *Tg(RHO P347S)*; *Edn2^−/−^* retinas compared to *Tg(RHO P347S); Edn2^+/+^* retinas (Fig. 7A). We also quantified the expression of *Gm12541* because the microarray analyses indicated that the dramatic increase in *Gm12541* expression in *Tg(RHO P347S); Edn2^+/+^* retinas was reduced in *Tg(RHO P347S)*; *Edn2^−/−^* retinas (Fig. 6). The qRT-PCR analyses confirmed remarkable increases in the expression of *Edn2* and *Gm12541* in *Tg(RHO P347S)*; *Edn2^+/+^* vs. *Edn2^+/+^* retinas (70.3- and 29.8-fold respectively), as well as a substantial increase in *Fgf2* and *Gfap* expression (3.0- and 2.0-fold respectively; Fig. 7A). In the absence of EDN2 in *Tg(RHO P347S)*; *Edn2^−/−^* retinas, the expression of *Gm12541*, *Fgf2* and *Gfap* decreased significantly (from 1.6–2.6 fold;Fig. 7A).

We then used FGF immunostaining to ask whether expression of the FGF2 protein was also increased in *Tg(RHO P347S)* retinas, in the presence and absence of EDN2, and whether the increase occurred in the mutant PRs or other retinal cell types (Fig. 7B). Low levels of FGF2 expression were detectable in all three nuclear layers of both *Edn2^+/+^* and *Edn2^−/−^* retinas (Fig. 7B). In *Tg(RHO P347S); Edn2^+/+^* retinas, FGF2 expression was strongly up-regulated, predominantly in PRs (Fig. 7B). In contrast, the increase in the PR FGF2 expression was abolished in the *Tg(RHO P347S)*; *Edn2^−/−^* retina (Fig. 7B), demonstrating that EDN2 directly or indirectly regulates the induction of PR FGF2 expression induced by the *Tg(RHO P347S)* mutation. Altogether, these findings establish that the increased expression of both the *Fgf2* mRNA and the FGF2 protein in the mutant retina are largely dependent on the expression of EDN2.

An increase in the Müller cell expression of GFAP was also observed in the *Tg(RHO P347S); Edn2^+/+^* retina, as has been reported in other IPDs [19], [46] (Fig. 7B). GFAP expression in Müller cells decreased significantly in the *Tg(RHO P347S)*; *Edn2^−/−^* retina, although not entirely, indicating that the expression of GFAP in Müller cells in the mutant retina is partly regulated, directly or indirectly, by EDN2 (Fig. 7B).

## Discussion

### Increased PR *Edn2* expression appears to be a survival response

We have shown that *Edn2* mRNA is induced in *Prph2^rds/+^*, *Tg(RHO P347S)*, and *Pde6b^rd1/rd1^* retinas at early stages of mutant PR degeneration. This finding, combined with those of Rattner and Nathans [19], and others [5], [47], establish that the greatly increased expression of *Edn2* is a common and possibly general response of PRs (including cones [5]) to the presence of an IPD mutation in either the PRs or in the RPE [21], and is elicited even though the mutations affect proteins with widely diverse functions. The increased PR expression of *Edn2* appears to be an early response imposed by a mutation [19] or other insults, including mechanical and light damage [19], [20], rather than an event in a death-signaling cascade, because the increase occurs early in mutant PRs, the majority of which will not die for weeks to months after birth, at least in the *Prph2^rds/+^* and *Tg(RHO P347S)* models.

Using the *Pde6b^rd1/rd1^* model mouse, we have presented two lines of evidence that the increased expression of *Edn2* in mutant PRs is a survival response. First, augmentation of the endogenous increase in *Edn2* expression in the *Pde6b^rd1/rd1^* mouse with the AAV-mat*Edn2* cDNA vector improved PR survival, by 31% between PN8 and PN15. Second, the restoration of *Edn2* expression with either the AAV-mat*Edn2* or AAV-prepro*Edn2* vectors enhanced PR survival in *Pde6b^rd1/rd1^*; *Edn2^−/−^* retinas by 14–18%. Although not large, this latter increase is notable, because it is additive to the enhanced PR survival that we identified in the systemic absence of EDN2 function, in *Pde6b^rd1/rd1^*; *Edn2^−/−^* mice *in vivo*. The increased survival are also notable given that the rate of PR death in *Pde6b^rd1/rd1^* retinas is the most rapid of all IPD models, with the great majority of PRs being ablated by PN21 [48].

Although the 31% increase in PR survival in *Pde6b^rd1/rd1^* mice observed using the AAV-mat*Edn2* cDNA vector is modest, we suggest that this finding is important. First, it provides the initial evidence that *Edn2* gene transfer can improve mutant PR survival. Second, the rate of PR death in the *Pde6b^rd1/rd1^* retina is much more rapid than any other mouse model known; the biological insult to the PR is extreme.

The improved PR survival observed with AAV-*Edn2* gene transfer suggests future studies to determine if even greater survival can be obtained by additional enhancement of the EDN2 signaling pathway. For example, since the conversion of prepro EDN2 to mat EDN2 by ECEs may be the rate-limiting step in controlling the availability of EDN2, AAV-mediated transfer of the ECE-1 cDNA may increase mutant PR survival. The possibility that ECE-mediated cleavage of big EDN2 (Fig. 4B) is the rate-limiting step in the formation of EDN2 is supported by the finding of Telemaque *et al*. [49], who found that increased systemic blood pressure following adenovirus-mediated over-expression of prepro EDN1 required the co-injection of an ECE-1 expression construct.

Other strategies to augment EDN2 signaling should also be examined, such as the use of agonists of the EDN2 signaling pathway to increase the response to EDN2. For example, the administration of an EDNRB agonist in light damaged retinas has been shown to reduce the number of dying cells [5].

### A role for *Edn2*-dependent increased FGF2 expression in mutant PR survival

The mechanism by which EDN2 mediates its protective effect in mutant PRs remain to be elucidated. One starting point for mechanistic analysis will be to define the roles of the 20 genes we found to have a >4-fold increased expression in *Tg(RHO P347S)* retinas, including *Fgf2*. We have shown that the increased expression of *Fgf2* may be EDN2-dependent. First, we found that *Fgf2* mRNA in whole retina is 3-fold increased in *Tg(RHO P347S)* mice compared to WT mice. This increase did not occur in the absence of EDN2 in *Tg(RHO P347S)*; *Edn2^−/−^* retinas. Second, we demonstrated, that FGF2 immunolabelling was increased in *Tg(RHO P347S)* PRs, but not in the absence of EDN2 in *Tg(RHO P347S)*; *Edn2^−/−^* PRs.

Other evidence supports our proposal that the protective effect of increased *Edn2* expression in mutant PRs is due, at least in part, to the *Edn2*-dependent increase in the expression of PR FGF2. First, exogenous FGF2 is an established survival factor for mutant PRs [9], [45]. Second, like *Edn2*, retinal *Fgf2* transcripts are induced under conditions of both genetic and environmental PR injury [50]. Third, *Fgf2* mRNA [51] and FGF2 protein expression is increased in Müller cells [52] in models of PR injury, and increased FGF2 immunofluorescence has been observed in PR cell bodies after optic nerve section. This increase may represent increased FGF2 expression, increased binding of FGF2 to FGFR1 [53], or both. Fourth, intravitreal administration of an EDNRB agonist increases *Fgf2* mRNA levels in whole WT retina, also consistent with FGF2 being part of an EDN2/EDNRB signaling pathway [5]. Finally, EDN1, which exhibits the same selectivity of binding to endothelin receptors as EDN2, is known to induce *Fgf2* mRNA expression in vascular smooth muscle cells [54]. Our data highlights a putative EDN2/EDNRB/FGF2 signaling pathway that may be downstream of leukemia inhibitory factor (LIF)-activated signals. A key role for LIF has been identified in PR survival in response to retinal insult. LIF was found to be necessary for the up-regulation of the *Edn2* and *Fgf2* mRNAs and for increased PR survival in both rhodopsin VPP mutant retinas [5] and light-damaged retinas [4]. Furthermore, intravitreal injection of recombinant LIF into *Lif^−/−^* retinas strongly induced the expression of *Edn2*. Since *Lif* is expressed in a subset of Müller glia in response to PR injury [5], we propose that one PR survival pathway may involve Müller cell-derived LIF induction of PR EDN2, which then binds to EDNRB on Müller cells; an as yet unidentified Müller cell signal then acts on the PRs to increase FGF2 expression. LIF signaling is mediated through gp130, and PR gp130 has been shown to contribute to PR survival in VPP retinas [55], indicating that PR gp130 may also be a component of the EDN2/EDNRB signaling pathway. Recent work by Braunger et al. suggests that EDN2 may participate in more than one PR survival pathway, depending on the upstream stimulus. In a light damage model, PR protection mediated by the over-expression of Norrin, a Wnt/B-catenin signaling molecule, was also found to be dependent on EDN2/EDNRB signaling [33]. In this model, however, the authors concluded that a LIF/EDN2/FGF2 signaling pathway was likely not associated with the protective effects against light damage observed in Rpe65-Norrin-2 transgenic animals. Taken together, the data suggest that EDN2 signaling may participate in the PR response to retinal injury via multiple survival pathways, including a LIF-independent Norrin-activated pathway and a LIF- and FGF2-dependent pathway.

It is noteworthy that three of the genes whose expression decreased the most in *Tg(RHO P347S)* retinas in the absence of EDN2: *Fgf2*, *Gm12541* and *Nudt6*, are all located and overlap on mouse chromosome 3. *Fgf2* is sandwiched between *Gm12541* and *Nudt6*, which are transcribed from the opposite DNA strand from *Fgf2*. *Gm12541* overlaps the coding region of *Fgf2* at its 5′ end, and *Nudt6* overlaps the 3′ UTR of *Fgf2* at its 3′ end. This arrangement, together with the coordinate expression of these three genes in the *Tg(RHO P347S)* retina, suggests that regulatory relationships may exist between them. Indeed, the mRNA of *Nudt6* is a known *Fgf2* antisense transcript (*Fgf2-AS*) that inhibits *Fgf2* expression [56], [57]. The *Gm12541* gene predicts a hypothetical protein, but it may also be a non-coding RNA that acts as an *Fgf2* regulatory antisense transcript. Some natural antisense transcripts regulate their sense partners positively, whereas others are negative regulators [58], [59]. A variety of regulatory models involving *Fgf2, Nudt6* and *Gm12541* could account for our finding that the expression of these three genes is coordinately regulated in the *Tg(RHO P347S)* retina.

### Systemic loss of EDN2 function also increases the survival of mutant PRs

In addition to the enhanced PR survival observed with AAV-mediated transfer of the mat*Edn2* gene to mutant PRs, we also found that systemic loss of EDN2 function was also protective to PRs. We propose that the increased PR survival in *Pde6b^rd1/rd1^*; *Edn2^−/−^* retinas *in vivo* (vs. in explants) is not due to the loss of PR *Edn2* expression from mutant PRs because i) the restoration of PR *Edn2* expression using either the AAV-mat*Edn2* or AAV-prepro*Edn2* vectors enhanced PR survival in *Pde6b^rd1/rd1^*; *Edn2^−/−^* mice, rather than accelerating PR death; and ii) PR survival was not increased in *Pde6b^rd1/rd1^*; *Edn2^−/−^* retinal explants, consistent with the increased survival observed in *Pde6b^rd1/rd1^*; *Edn2^−/−^* retinas *in vivo* being due to extraocular mechanisms.

We propose that the pulmonary abnormalities and consequent systemic hypoxia of *Edn2^−/−^* mice (unpublished data), perhaps together with other changes due to the systemic loss of *Edn2* expression, may be responsible for the increased *in vivo* PR survival in *Pde6b^rd1/rd1^*; *Edn2^−/−^* and *Tg(RHO P347S)*; *Edn2^−/−^* mutant retinas. A role for hypoxia in the increased PR survival is supported by our finding of increased serum EPO and increased retinal VEGF levels in *Edn2^−/−^* mice, and by our previous demonstration that the maintenance of *Pde6b^rd1/rd1^* retinal explants in 6% O_2_ increased PR survival [42]. Moreover, Grimm et al. [60] found that preconditioning mice in 6% O_2_ up-regulated retinal EPO, VEGF and FGF-2 and fully protected PRs from light damage. In addition, systemic EPO administration has been shown to increase PR survival in response to both light damage or IPD mutations [60], [61]. Increased retinal VEGF expression may also contribute to the increased PR survival observed with hypoxia, since VEGF was found to be protective of *ex vivo* retinal cultures in a model of ischemia-reperfusion injury [62], and to reduce PR apoptosis in isolated PR cell and outer nuclear layer explants [63]. Altogether, these findings suggest that the >60% increase in PR survival that we observed in *Tg(RHO P347S)*; *Edn2^−/−^* and *Pde6b^rd1/rd1^*; *Edn2^−/−^* mice *in vivo* may be the result, at least in part, of retinal hypoxia and the attendant increases in systemic EPO and intraocular VEGF.

### Therapeutic implications of the common *Edn2* response in IPDs

We previously demonstrated that, like the increase in *Edn2* expression in mutant PRs, a diverse set of IPDs display another common behavior, exponential kinetics of PR death [64], [65]. We proposed that the shared kinetics suggest a commonality in the biochemical responses of mutant PRs to a mutation. The biochemical changes that occur downstream of a PR mutation may converge on a relatively small number of shared pre-apoptotic biochemical pathways that resist or mediate PR death [65]. The increase in PR *Edn2* expression in multiple models of IPD would appear to be one such shared downstream biochemical response. Consequently, therapies to enhance EDN2 signaling may have potential as a general therapy for IPDs, irrespective of the gene affected.

Other animal models of IPD must now be examined, to determine if AAV-mat*Edn2* gene transfer increases mutant PR survival in additional models, and whether the increase is greater with less severe mutational insults than that which occurs in the *Pde6b^rd1/rd1^* retina. In addition, tests must be undertaken to establish that the improved PR survival seen with AAV-mat*Edn2* gene transfer is accompanied by conservation of vision, and that long-term increased *Edn2* expression does not have adverse effects on retinal function or survival. The development of general therapies that are safe and effective in a broad range of animal models of IPDs has important potential for patients for whom a gene-specific therapy has not yet been developed.

Finally, our studies add to the principle, as shown with FGF2 therapy for IPDs [9], [10], that augmentation of the endogenous survival responses of mutant PRs merits on-going examination to identify potential general therapies for this debilitating group of monogenic diseases.

## Materials and Methods

### Ethics Statement

Animals were raised under 12-hr/light dark cycles and treated in accordance with the guidelines and principles outlined by the Animal Care Committee at the Hospital for Sick Children (Toronto, ON).

### Mice

Mice carrying the *Pde6b^rd1/rd1^* (C3H/HeOuJ) and *Prph2^rds/rds^* (C3A.BLiA-Pde6b^+^.O20-Prph2^Rd2^/J) alleles, including *Prph2^+/+^* (C3A.BLiA-Pde6b^+^/J) and C57BL/6J controls were obtained from The Jackson Laboratory (Bar Harbor, ME). *Prph2^rds/+^* mice were generated by crossing *Prph2^rds/rds^* and *Prph2^+/+^* mice. *Tg(RHO P347S)* mice were obtained as a gift from Dr. Tiansen Li (Boston, MA). EDN2 null mice (129Sv/Ev Taconic) were generated by replacing part of exon 1 and all of exon 2 (corresponding to mature EDN2), with a Neo cassette. Unflavored Peptamen (a complete elemental nutritional supplement; Nestle, North York, ON) was given to EDN2 mull mice to improve survival.

### RNA preparation, cDNA synthesis and qRT-PCR

Total retinal RNA was extracted with Trizol (Invitrogen, Burlington, ON) and purified using the RNeasy Mini kit (Qiagen, Mississauga, ON) with on-column DNA digestion using the RNase-free DNase set (Qiagen). Purified RNA was resuspended in 30 µl RNase-free ddH2O and cDNA generated using the Omniscript Reverse Transcription Kit (Qiagen). qRT-PCR was performed using the ABI Prism® 7900HT sequence detection system (Applied Biosystems, Foster City, CA), and Sequence Detector System 2.2.1 software (Applied Biosystems) was used to analyze the data.

### HPLC, RIA and ELISA

Control peptides (EDN1, EDN2, and EDN3) were obtained from Peptides International (Louisville, KY). Retention times and peak areas were recorded on a tracer (BBC Goerz Metrawatt, Sevigor 120, Markham, ON). Samples were chloroform: methanol extracted and run through a C-2 column (GE Healthcare, QC) before lyophilization. Isolation of EDN2 was performed using a Zorbax SB-C18 HPLC column (5 micron, 300Å pore size, Agilent technologies, Mississauga, ON) and the peptide was quantified using an Endothelin RIA kit (RPA 555, GE Healthcare). Retinal VEGF was assayed using the mouse VEGF Quantikine ELISA kit (MMV00, R&D Systems, MN) and serum EPO using the Mouse/Rat EPO Quantikine ELISA kit (MEP00, R&D Systems).

### *In situ* hybridization, histology and immunostaining

*In situ* hybridization was performed using digoxygenin-labeled riboprobes transcribed from cloned PCR products as previously described [19]. For frozen sections, eyes were enucleated, fresh frozen in OCT compound (Tissue-Tek, Miles, Elkhart, IN), and 10 µM sections prepared. Sections were post-fixed in 4% paraformaldehyde (GFP) or ice-cold acetone (EDNRA, EDNRB) and blocked with 5% normal goat serum. Sections were incubated with rabbit anti-EDNRA (1∶100, AER-001, Alomone Labs, Jerusalem, Israel), anti-EDNRB (1∶100, AER-002, Alomone Labs, Jerusalem, Israel) or rabbit anti-GFP (1∶1000, ab290, Abcam, Cambridge, MA) followed by goat anti-rabbit secondary Alexa488 (1∶500, Invitrogen).

For paraffin sections, eyes were fixed with 4% paraformaldehyde solution. 7 µM sections were cut and deparaffinized in xylene. Antigen retrieval was performed with 0.05% trypsin for 15 min at 37°C (ECE-1) or incubation for 20 min at boiling in sodium citrate buffer solution (10 mM Sodium Citrate, 0.05% Tween 20, pH 6.0) (GS, GFAP, FGF2). Sections were either stained with H&E or blocked with 5% normal goat serum and incubated with anti-GS (1∶5000, G2781, Sigma-Aldrich, Oakville, ON), anti-ECE-1 (1∶100, AP6855b, Abgent, Brockville, ON), anti-GFAP (1∶500, G9269, Sigma-Aldrich) or FGF2 (1∶400, ab106245, Abcam). Fluorescence was visualized with Alexa488 secondary antibodies (1∶500, Invitrogen).

For histology of retinal explants, retinas were fixed in 4% paraformaldehyde for 2 hours at RT, embedded in OCT compound medium (Tissue-Tek), and stained with H&E.

### Retinal ONL measurements

Enucleated eyes were marked with a flamed needle to identify the superior (dorsal) hemisphere and fixed in 2% gluteraldehyde/0.1 M phosphate buffer. Superior and inferior globes were cut into three equivalent parts and the rostral (superior) and caudal (inferior) slices of the nasal quadrants embedded in Jembed 812 (Canemco, Lakefield, QC). 800 nm sections taken from nasal rostral and caudal quadrants were cut and stained with 1% toluidine blue. The width of the ONL was measured using SigmaScan Pro (Systat Software Inc., San Jose, California). To preclude variations in local retinal degeneration rates from influencing ONL measurements, ONL thickness was determined in orthogonal sections, by measuring thickness at a point 25% of the distance from central to peripheral retina in *Pde6b^rd1/rd1^*mice, and at 75% of this distance in *Tg(RHO P347S)* retinas. The % change in ONL thickness as indicated refers to (|M−N|/N×100%), where N is the normal condition and M can indicate the loss of EDN2 or introduction of the *Edn2* cDNA vector.

### Retinal Explants

Mice were decapitated at PN7 and retinal explants with attached retinal pigment epithelium (RPE) were obtained as previously described [41]. Eyes were incubated in proteinase K solution (1.2 mg/mL) (Roche, Laval, Quebec) for 15–20 minutes at 37°C to separate the sclera and RPE, and dissected retinas were laid flat on Millicell-HA 0.45 µM filter membranes (Millipore, Billera, CA). Explants were maintained under serum and antibiotic-free conditions in R16 basal culture medium (Invitrogen, CA). Media was changed every two days.

### AAV production and subretinal delivery

Self-complementary AAV vectors containing the small, hybrid cytomegalovirus-chicken β-actin (smCBA) promoter driving expression of either *Gfp*, prepro*Edn2* or mat*Edn2* were generated and purified by previously described methods [66]. Vector titer was determined by qRT-PCR and final aliquots were resuspended in balanced salt solution (Alcon Laboratories, Forth Worth TX, USA) containing 0.014% Tween 20. qRT-PCR expression of the AAV-specific *Edn2* mRNA was measured using a 3′primer specific to the 3′ tail of the mRNA, including the polyA sequence.

For subretinal injections, mice were anaesthetized with ketamine/xylazine (IP, 150 mg/kg ketamine, 10 mg/kg xylazine) and the cornea punctured with a 30-gauge needle between the corneoscleral junction and the ora serrata. A 33-gauge blunt needle attached to a 10 µl Hamilton syringe (Hamilton Company, Reno, NV) was used to dispense 1 µl of fluid (viral titre 1–5×10^12^ VG/mL) containing 0.1 mg/mL fluorescein (Alcon, Mississauga, ON) to aid visualization of the subretinal injection site. Mice were treated with topical tropicamide ointment (Alcon) following injection and administered 300 µl normal saline S.C. to prevent dehydration.

### Microarrays

For microarray studies, retinas were homogenized in GIT buffer (4 M guanidine isothiocyanate, 25 mM sodium acetate (pH 6), 120 mM β-mercaptoethanol). Total RNA was further isolated by cesium chloride density gradient centrifugation and purified using the RNeasy Mini kit (Qiagen). RNA quality was assessed using a Bioanalyzer (Agilent Technologies, Mississauga, ON). Briefly, labeled cRNA was hybridized to the Mouse 430_2.0 Array representing over 39,000 mouse transcripts and probe sequences from GenBank®, dbEST, and RefSeq (Affymetrix, Santa Clara, CA). Affymetrix Gene Chip Operating Software (GCOS) was then used to generate.cel microarray files which were further analyzed using Robust Multi-chip Averaging (RMA) algorithms (Affymetrix). Significance analysis was performed using the Significance Analysis of Microarrays program (SAM, Stanford University Labs) [67], and a list of differentially expressed genes generated. For the *Edn2^+/+^* vs. *Tg(RHO P347S) Edn2^+/+^* and *Edn2^−/−^* vs. *Tg(RHO P347S) Edn2^−/−^* comparisons, an FDR of 5% was used, whereas for the *Edn2^+/+^* vs. *Edn2^−/−^* analysis, an FDR of 25% was used (NCBI GEO Accession GSE38797).

## Supporting Information

Figure S1 **Quantification of mRNA expression of** ***Edn1, Edn3, EdnrA*** **and** ***EdnrB*** **in mouse retinas.** qRT-PCR was used to quantify the expression of *Edn1*, *Edn3*, *EdnrA* and *EdnrB* in *Tg(RHO P347S)* retinas in the presence and absence of EDN2 function. Bar graphs show the expression of mRNAs in retinas of the indicated genotypes relative to the expression levels seen in *Edn2^+/+^* (WT) retinas (arbitrarily assigned a value of 1). There were no significant differences in expression except for a slight increase in the *EdnrA* mRNA in *Tg(RHO P347S);Edn2^−/−^* vs. *Edn2^+/+^* retinas (^*^n = 3, p<0.05) (n = 3;p>0.05 for all other comparisons). All qRT-PCR values were normalized to *Gapdh* mRNA.
(TIF)

Figure S2 **Immunostaining of ECE-1, EDNRA and EDNRB in mouse retinas.** (A) The localization of ECE-1 immunofluorescence in WT retina overlaps that of the Müller cell marker glutamine synthetase. ECE-1 expression was observed in both Müller cell extensions and cell bodies. (B) Immunofluorescence localization of EDNRA and EDNRB in mouse retinas. EDNRA staining was sporadically observed in the GCL, INL, OPL and choroid plexus, irrespective of genotype. Although EDNRA staining in choroid, GCL and OPL is indicative of vessels [26], EDNRA expression in the INL and ONL of *Pde6b^rd1/rd1^*; *Edn2^−/−^* retinas may represent retinal microglia (42), which migrate to the mutant PRs in the ONL [39]. EDNRB immunoreactivity was predominantly observed in Müller cell radial fibres and in the OPL and GCL, possibly representing horizontal cells and astrocytes, respectively [25]. In the *Pde6b^rd1/rd1^* retina, EDNRB expression was significantly increased in Müller cell radial fibres, with stronger staining in the inner limiting membrane; this stronger staining may correspond to the end feet of Müller cells as well as astrocytes [69]. OLM, outer limiting membrane; ONL, outer nuclear layer; OPL, outer plexiform layer; INL, inner nuclear layer; GCL, ganglion cell layer; ILM, inner limiting membrane. (Bar = 25 µm).
(TIF)
