# Supplementary figures and images for: Endothelin-2-Mediated Protection of Mutant Photoreceptors in Inherited Photoreceptor Degeneration
Source: PLoS One. 2013 Feb 28;8(2):e58023. doi: 10.1371/journal.pone.0058023 (PMC3585171; doi:10.1371/journal.pone.0058023)

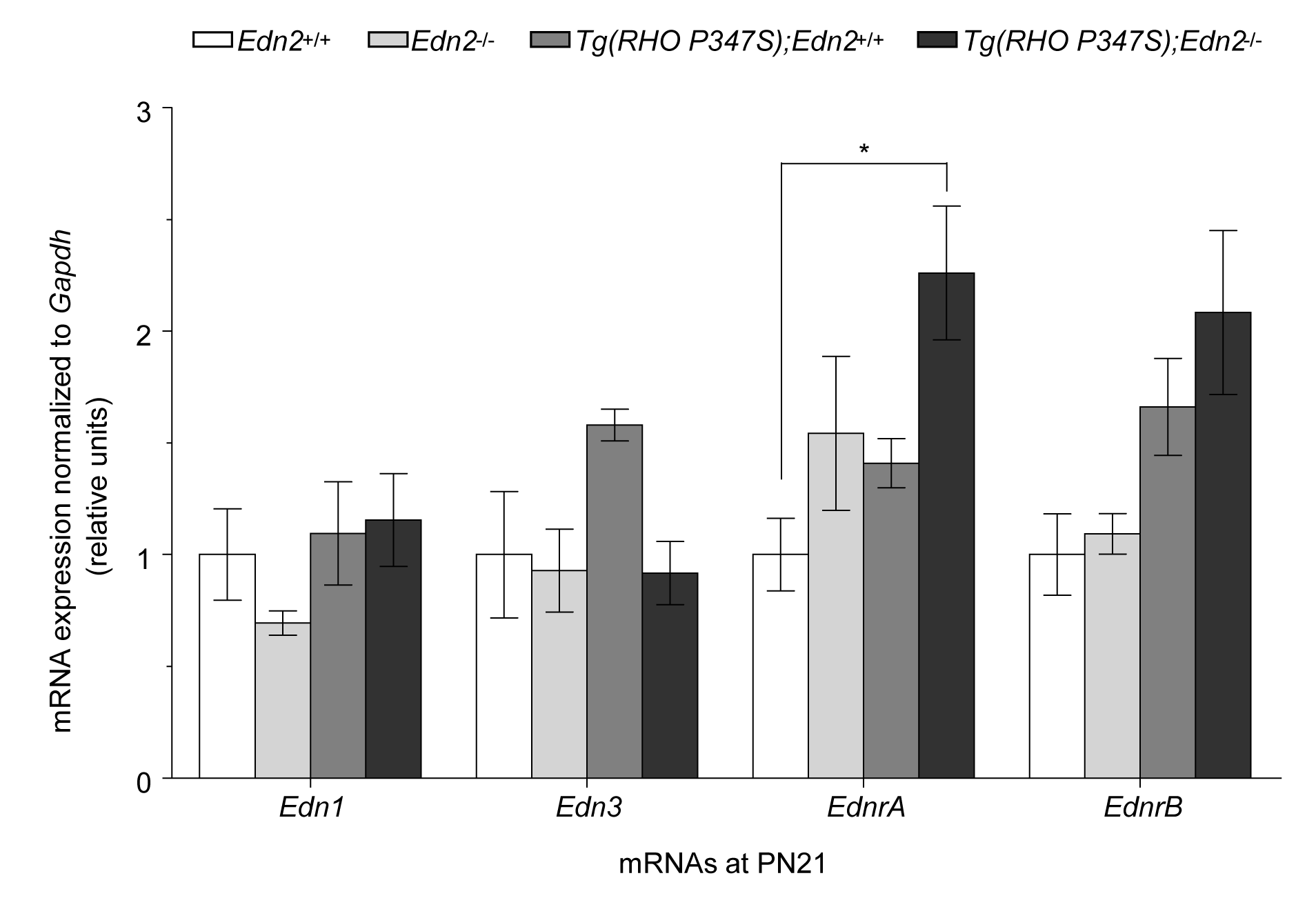

Supplement: Figure S1 — Quantification of mRNA expression of Edn1, Edn3, EdnrA and EdnrB in mouse retinas. qRT-PCR was used to quantify the expression of Edn1, Edn3, EdnrA and EdnrB in Tg(RHO P347S) retinas in the presence and absence of EDN2 function. Bar graphs show the expression of mRNAs in retinas of the indicated genotypes relative to the expression levels seen in Edn2+/+ (WT) retinas (arbitrarily assigned a value of 1). There were no significant differences in expression except for a slight increase in the EdnrA mRNA in Tg(RHO P347S);Edn2−/− vs. Edn2+/+ retinas (*n = 3, p<0.05) (n = 3;p>0.05 for all other comparisons). All qRT-PCR values were normalized to Gapdh mRNA. (TIF) [file pone.0058023.s001.tif]

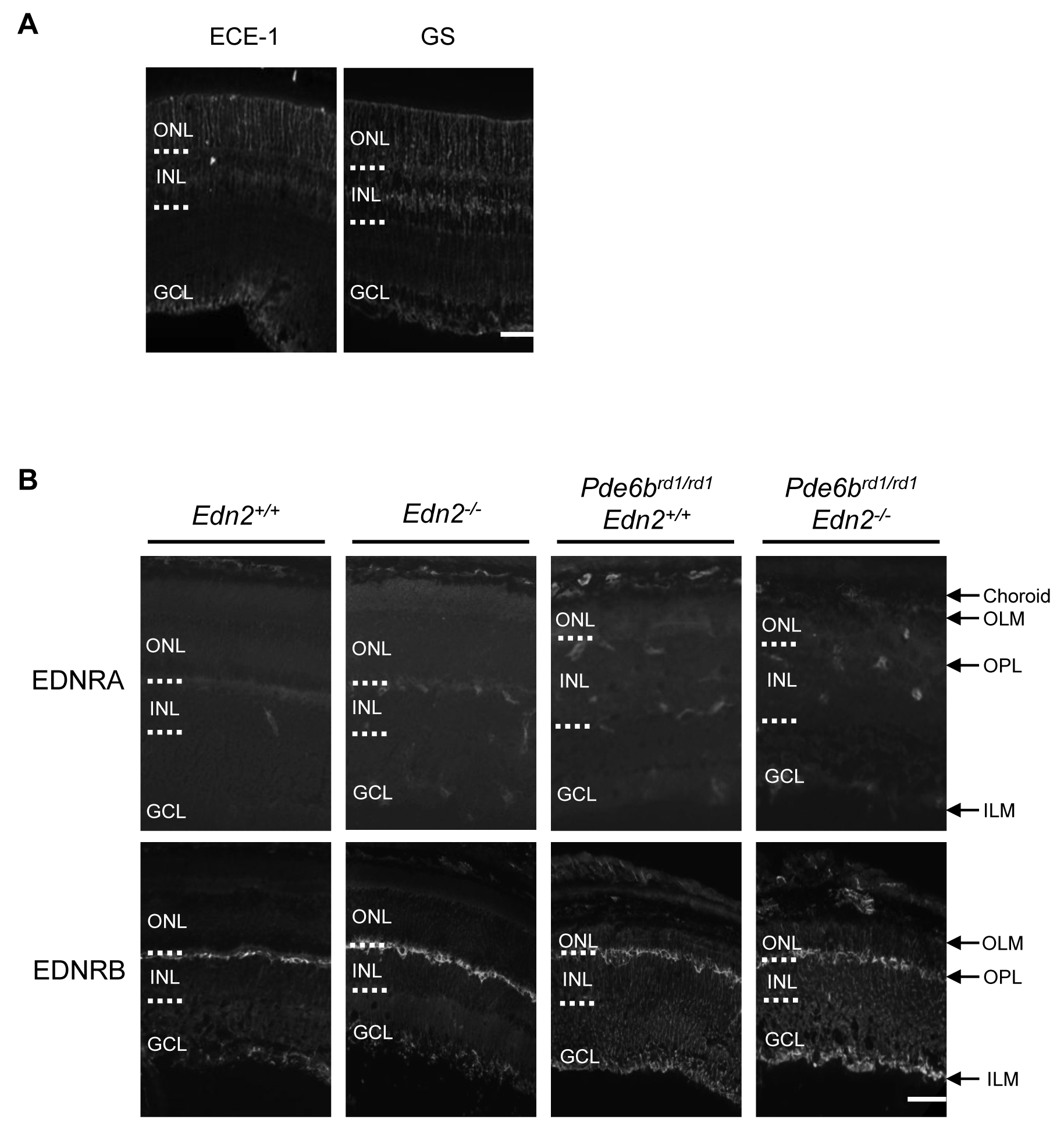

Supplement: Figure S2 — Immunostaining of ECE-1, EDNRA and EDNRB in mouse retinas. (A) The localization of ECE-1 immunofluorescence in WT retina overlaps that of the Müller cell marker glutamine synthetase. ECE-1 expression was observed in both Müller cell extensions and cell bodies. (B) Immunofluorescence localization of EDNRA and EDNRB in mouse retinas. EDNRA staining was sporadically observed in the GCL, INL, OPL and choroid plexus, irrespective of genotype. Although EDNRA staining in choroid, GCL and OPL is indicative of vessels [26], EDNRA expression in the INL and ONL of Pde6brd1/rd1; Edn2−/− retinas may represent retinal microglia (42), which migrate to the mutant PRs in the ONL [39]. EDNRB immunoreactivity was predominantly observed in Müller cell radial fibres and in the OPL and GCL, possibly representing horizontal cells and astrocytes, respectively [25]. In the Pde6brd1/rd1 retina, EDNRB expression was significantly increased in Müller cell radial fibres, with stronger staining in the inner limiting membrane; this stronger staining may correspond to the end feet of Müller cells as well as astrocytes [69]. OLM, outer limiting membrane; ONL, outer nuclear layer; OPL, outer plexiform layer; INL, inner nuclear layer; GCL, ganglion cell layer; ILM, inner limiting membrane. (Bar = 25 µm). (TIF) [file pone.0058023.s002.tif]
